# Current Practices and Recommendations for Children with Food Allergies and Feeding Behaviours: Insights from a Survey Among Australian Health Professionals

**DOI:** 10.3390/children12070905

**Published:** 2025-07-09

**Authors:** Jennifer Kefford, Rebecca L. Packer, Merryn Netting, Elizabeth C. Ward, Jeanne Marshall

**Affiliations:** 1 School of Health and Rehabilitation Sciences, The University of Queensland, St Lucia, Brisbane, QLD 4072, Australia; rebecca.packer@uq.edu.au (R.L.P.); liz.ward@uq.edu.au (E.C.W.); jeanne.marshall@health.qld.gov.au (J.M.); 2 Child Youth and Family Health Service, Primary and Community Health, Northern Sydney Local Health District, NSW Health, Sydney, NSW 2000, Australia; 3 Department of Paediatrics, University of Adelaide, Adelaide, SA 5005, Australia; merryn@nationalallergy.org.au; 4 National Allergy Council, Sydney, NSW 2000, Australia; 5 Centre for Functioning and Health Research, Queensland Health, Brisbane, QLD 4102, Australia; 6 Queensland Children’s Hospital, Children’s Health Queensland Hospital and Health Service, Brisbane, QLD 4101, Australia

**Keywords:** food allergy, paediatric feeding disorder, service delivery, health professionals

## Abstract

**Background**: Children with food allergies can present with paediatric feeding disorder (PFD). However, access to coordinated multidisciplinary services to support these children in Australia is inconsistent. To date, the availability of services or the perceived care needs of Australian health professionals working with this population have not been formally explored. **Methods**: A web-based survey was distributed to health professionals in Australia. Quantitative demographic data were summarised using descriptive statistics, and open-ended responses were analysed using content analysis. **Results**: The final sample comprised 98 responses, with speech pathologists representing the largest professional group (*n* = 39; 40%). A majority (59%) worked in hospital-based services. Open-ended responses were coded utilising content analysis. Three categories were developed including (1) service delivery, (2) intervention, and (3) resources. Services were commonly impacted by long wait times, limited staff training, and inconsistencies between hospital and community care. Additionally, mental health support was frequently reported as insufficient. **Conclusions**: The findings from this study underscore the need for integrated services for children with food allergies and paediatric feeding disorder. Recommended areas for future research include exploring caregiver perspectives and the impact of food allergies and paediatric feeding disorder, and consideration of co-designed studies to inform service improvement initiatives.

## 1. Introduction

Food allergies affect between 5 and 10% of young children worldwide [1,2,3], and around 10% of children in Australia present with food allergy by the age of 12 months [4]. There is no cure for food allergies and the only management is food allergen avoidance [5]. However, there is now increased recognition that food allergies have a wide impact on many aspects of a child’s daily life, which also require management. Netting, McWilliam [6] described how a child’s nutrition, growth, feeding development, feeding dynamic between caregiver and child, and a child’s dietary patterns can be affected by food allergy. Psychological impacts including bullying, caregiver and child anxieties around living with food allergies, and unnecessarily restricted diets have also been reported [7]. In addition, children with food allergies have lower health-related quality of life than children without food allergies with restrictions on social participation being one of the largest impacts [8].

In addition to impacts on health and development, young children with food allergies may present with characteristics of paediatric feeding disorder [9,10,11,12]. Paediatric feeding disorder is a diagnostic term applied when a child presents with impaired oral intake that is not considered age-appropriate, associated with medical, nutritional, feeding skills and/or psychosocial dysfunction [13]. The general prevalence of paediatric feeding disorder has been estimated to be between 1 in 23 to 1 in 37 children [14]. For children with food allergies, it is generally hypothesised that food introduction is disrupted due to adverse reactions to food allergen/s, and their diet is medically restricted to avoid these triggers [6]. This can result in further restriction from the child as they refuse to eat due to fear of further reactions or learned aversion [15]. This is particularly an issue for children with delayed diagnosis of food allergy or those who have multiple exposures to their food allergen.

Caregiver anxiety around potential allergen exposure can also lead to a child being fed the same few foods repetitively, which may impact or delay oral skills and increase food neophobia. A recent scoping review explored the literature regarding characteristics of feeding disorder observed in children with food allergies, concluding that there is a complex interplay between these two conditions [9]. Common characteristics observed included food refusal and aversion, anxiety with eating, poor intake, slow eating, and delays in oral sensory motor skills [9]. Kefford and colleagues [9] asserted that better characterisation of paediatric feeding disorder in this population may support earlier intervention and help mitigate the negative impact of allergen-related aversive experiences on feeding development.

Childhood allergies and paediatric feeding disorder are complex and require multidisciplinary, family-centred care, with access to health professionals such as allergists/clinical immunologists, paediatricians, speech pathologists, dietitians, occupational therapists, and psychologists [16,17,18,19]. Recently, the European Academy of Allergy and Clinical Immunology (EAACI) task force called for integrated, multidisciplinary management of food allergies [16,20]. Unfortunately, to date, international research in paediatric food allergy care highlights that there is often a considerable wait time for services, causing delays in diagnosis, inhibiting timely access to early intervention, and impeding ongoing management [21,22,23]. This challenge is echoed in paediatric feeding disorder care, where appropriate and timely access to services has been identified as an issue [24,25,26,27,28], and little is known about whether available services are multidisciplinary. Health professionals’ knowledge regarding practice in both of these areas has also been identified as lacking, with many requesting more training in both food allergies [16,20,21] and paediatric feeding disorder [27,29,30] due to the complexities involved in providing care for these children.

Despite research to suggest there is a significant relationship between food allergies and paediatric feeding disorder, and the importance of timely, multidisciplinary care, there is limited information regarding existing medical and allied health services in Australia for children who present with these conditions. The aim of this current study was to gather information from Australian health professionals regarding perceptions of existing service delivery, as well as to develop recommendations for best practice care models.

## 2. Methods

Ethical approval was obtained through The University of Queensland (2022/HEC000249) on 13 September 2022. All participants provided digital informed consent before accessing the survey content. The methodology and results are reported according to the Checklist for Reporting Results of Internet E-Surveys (CHERRIES) [31] and the Standards for Reporting Qualitative Research [32].

### 2.1. Participant Recruitment and Eligibility

This survey was targeted at Australian health professionals who worked in settings that may have supported children with food allergies and paediatric feeding disorder. This included health professionals from the disciplines of speech pathology, nutrition and dietetics, occupational therapy, as well as early childhood nurses, paediatricians, allergists/clinical immunologists, general practitioners (GPs), gastroenterologists, and psychologists. Potential participants were invited to participate through online advertisements in the monthly e-newsletters distributed by Speech Pathology Australia, the National Allergy Centre of Excellence, the Australasian Society of Clinical Immunology and Allergy, and the Australian Paediatric Dysphagia Interest Group over a 9-month period. Participants were also encouraged to share the survey with colleagues (i.e., snowball sampling). Participants were advised that all responses were anonymous. No incentives were offered for participation.

### 2.2. Survey Development and Dissemination

The survey was purpose-built. The initial draft survey was developed by four authors (JK, RP, EW, JM), two of whom were speech pathologists with experience working with children with food allergies and paediatric feeding disorder. The survey contained 20 questions (15 multiple-choice and 5 open-ended questions). Multiple-choice questions were included to understand the participants and the context in which they managed children with food allergies and paediatric feeding disorder and covered: (a) general demographics of the participants (i.e., discipline, clinical work context, years of experience); (b) clinical caseload information (i.e., referral reason, common diagnoses, caregiver reported concerns); and (c) health service context. The second key component of the survey was a series of open-ended questions collected to examine participants’ perceptions of aspects of care for children with both food allergies and paediatric feeding disorder (see Supplementary Materials).

A cohort of eight medical and allied health professionals were then asked to pilot the initial survey and using a cognitive interviewing approach [33]. These eight health professionals were recruited through existing professional networks, ensuring the sample had diverse representation across disciplines, clinical experience, and geographical location. Three speech pathologists, two dietitians, one occupational therapist, one paediatric medical registrar, and one consultant paediatrician reviewed the survey and participated in a cognitive interview. The participants worked across three states, New South Wales (*n* = 4), Queensland (*n* = 3), and Western Australia (*n* = 1). Four participants had more than five years’ experience working with children with feeding concerns due to a range of aetiologies.

The cognitive interviews were conducted and recorded via Zoom^®^ by the first author (JK). The mean interview length was 18 min (range 9–34 min). During the cognitive interview process, the health professionals were asked to read each question live and then verbalise their thinking in response to the question. Data was collected on whether questions required repetition, clarification, or explanation. Any concerns raised regarding the clarity or variety of response options were recorded. These responses were summarised and then reviewed by two study authors (JK, JM). Questions with two or more suggestions or requests for clarification were discussed until consensus was reached on wording changes, additions, or deletions from the survey. Analyses identified that additional information was required for the definitions at the beginning of the survey to define terms such as “paediatric feeding disorder”, “feeding difficulties”, and “challenging feeding behaviours”. Clinical examples and a link to a national allergy website were added to define “food allergy”. On advice from participants, definitions were repeated halfway through the survey. Two questions were changed to allow multiple-choice answers, and four multiple-choice questions had additional choice options added. One question was moved to a different section to improve survey flow, and one question was added to better capture the clinical skill of participants. The final survey had 22 questions, with 17 multiple-choice and five open-ended questions (Supplementary Materials).

The final survey was delivered electronically via QualtricsXM and took approximately 20 min to complete online. The survey was open from February to November 2023. Participants were able to review their answers using a “Previous” button. Aside from the question regarding digital consent, no questions were mandatory, and there was no completeness check. Data was stored on the University of Queensland Research Data Manager system.

### 2.3. Data Analysis

Only surveys with >50% question completion were included in the final analysis. Quantitative data was reported using descriptive statistics in SPSS (Version 31), with responses reported as a proportion of the total number of participants. For the open-ended questions, content analysis [34] was used to identify common categories, subcategories and codes. Three authors (JK, JM, EW) reviewed the open-ended survey responses to generate codes and reach consensus on categories derived from the data. Any disagreements were discussed between these authors until consensus was reached. Quotes from free-text answers are included through the text but are anonymous. Frequencies of codes across different subcategories are reported through the Results Section.

## 3. Results

In total, 141 surveys were commenced, but 43 responses were excluded due to incomplete data, leaving 98 complete surveys included for analysis. Calculation of a total survey response rate was not feasible due to the indeterminate size of the population invited to participate, compounded by the addition of snowball sampling.

### 3.1. Participant Demographics

Demographics are detailed in Table 1. Most participants were speech pathologists (*n* = 39; 40%), and more were working in New South Wales, Australia (*n* = 52; 53%). There were no responses from psychologists or gastroenterologists, but all other disciplines targeted were represented. Most participants were working in a hospital setting (*n* = 58; 59%) with 25% (*n* = 25) from community health. The survey responses were mostly from experienced health professionals with 63% (*n* = 61) having over 10 years’ experience in paediatrics (Table 1).

### 3.2. Clinical Caseload Details

Participants’ caseload information is summarised in Table 2. Children under five comprised the majority of participants’ caseloads. Feeding concerns accounted for 10–50% of the caseload in 42% (*n* = 41) of participants, with 10–50% of these children estimated to have co-occurring food allergies. Common comorbidities included autism spectrum disorder (87%, *n* = 85), food allergies (84%, *n* = 82), and global developmental delay (73%, *n* = 72). The most frequently reported feeding concerns from caregivers were refusal to try new foods (77%, *n* = 76), self-restricted diets (70%, *n* = 68), and caregiver stress (67%, *n* = 65).

### 3.3. Service Inclusions

Participants described that staffing at their workplace included speech pathologists (75%, *n* = 74), dietitians (75%, *n* = 74), paediatricians (62%, *n* = 61), occupational therapists (58%, *n* = 57), and an allergist/clinical immunologist (44%, *n* = 44). No participant reported that a psychologist or social worker was available in their service. Participants reported that they could externally refer to a paediatrician (47%, *n* = 46), psychologist (42%, *n* = 41), GP (62%, *n* = 61), gastroenterologist (50%, *n* = 49), and allergist/clinical immunologist (50%, *n* = 49) for support with children with food allergy and/or feeding concerns. However, in free-text comments, many commented that access to medical specialists such as allergists/clinical immunologists required a GP referral, which limited their capacity to be referred directly. Participants who worked in a hospital more commonly reported access to a multidisciplinary feeding clinic than those not working in a hospital.

### 3.4. Factors Affecting Services for Children with Food Allergies and Feeding Concerns

Content analysis of the open-ended questions regarding factors affecting services for children with both food allergies and feeding concerns facilitated the development of three categories, including (1) service delivery, (2) intervention, and (3) resources. A summary of what participants perceived was being done well and what needed improvement in their service across these three categories is described below and summarised in Table 3.

### 3.5. Service Delivery

With regard to service delivery, there were various models of service provision described, such as “multidisciplinary team available” “integrated services”, “private feeding clinic”, community-based single discipline services with internal referral pathways, and sole private practitioners. One participant reported that a strength present in their facilities was the capacity to provide a “short-term intervention service until local services were in place”, but others reported that they were a “busy consultative service so [they offered] infrequent appointments, [it] would be better if we could offer more frequent reviews.” Others reported similar concerns, stating they were “only able to provide an assessment and limited short-term intervention service with limited ability to provide ongoing or long-term support”.

Another strength reported by some participants was being able to provide flexible modes of service delivery. Some were able to offer “flexible appointments—as regular as needed”, and others could offer “regular reviews based on need (face-to-face, phone, telehealth)”. Participants provided suggestions such as offering “MD [multidisciplinary] input with real world assessment in [the] home” as potential service options if they were able.

Providing comprehensive care was reported as a strength in a small number of services (*n* = 7) with the capacity to provide collaborative multidisciplinary sessions described by some (*n* = 13), and good links between allergy and feeding services reported by others (*n* = 3). However, this type of coordinated service was described as lacking by other participants (*n* = 6) that were not linked to any one setting. A large volume of participants (*n* = 71 comments) reported that in a perfect world, they would prefer a “collaborative service to manage children with food allergies and feeding concerns”, with access to “multidisciplinary and joint services to improve health outcomes”.

There were mixed views on communication both between teams managing these children and also between services. Some participants felt communication was a particular strength of their service, reporting that they were “seeking out information from allied health…and medical teams…in the forms of letters/reports and [were] taking this information into account when providing intervention.” Others reported that it was “extremely difficult to get health professionals who work on different directorates to be able to work collaboratively” and they had “challenges providing coordinated multidisciplinary care with medical/hospital teams (beyond report sharing)”. Participants also raised this concern in the perfect-world scenario, reporting that they wanted “notes shared between disciplines” and “links with community services to improve transition”.

Referral networks were another area of service delivery that showed contrasting views between hospital- and non-hospital-based services. Whilst participants from hospital settings generally reported good internal referral networks, with access to medical (*n* = 8) and allied health care (*n* = 13) commonly available, participants in non-hospital services reported difficulty making referrals to more local services for families and in a timely manner. One participant described the need for “stronger community networks so that there are more therapy options closer to home”. Participants also reported only “intermittent supports from visiting paediatricians”, “hardly ever [having] timely access to a full team”, and “LONG waiting times (12 months+)”. Another participant indicated a “lack of feeding services, both hospital- and community-based for children aged under 2 years if they don’t have an NDIS package”.

Various reasons were offered by the participants for these deficiencies, ranging from a lack of funding for positions, to working within a private single discipline practice, or being a rural clinician covering a large geographical area. Timely referrals were further highlighted in answers to the perfect-world scenario where participants wanted “timely access to paediatric allergists/immunologists/gastroenterologists” with “prompt investigation” and “more allied health availability/quicker appointments”.

### 3.6. Intervention

Several feeding intervention options were presented by participants as strengths in their service. The ability to provide “therapeutic support to children” through “hands-on and practical” sessions, and “having an in-house dietitian who works hand-in-hand with these families” were reported as examples of direct therapy being offered. One service reported they offered the Sequential Oral Sensory (SOS) Approach to Feeding [35], and another specified they followed responsive feeding practices.

By contrast, some participants reported that the provision of feeding intervention was a challenge for them as their “location does not support feeding therapy for the moment”. In the perfect-world scenario, many participants expressed the desire to offer direct and intensive therapy when needed, citing that they wanted to be able to offer “1:1 feeding therapy”, “feeding groups”, support for “mealtime management”, and “frequent therapy targeting challenging behaviours”.

With regard to intervention directed at caregivers, some participants (*n* = 5) reported that they were able to provide good caregiver support by “listening to the parent(s)” and “validating the families’ experiences”. Overall, however, supporting the mental health of caregivers in this population was highlighted as a significant concern. One participant stated that the priority for feeding therapy needs to “move to more focus on parents as the primary carer in relation to understanding their stress and how it impacts the feeding environment rather than so much therapy focus on the child”. Many participants also raised a desire for increased access to caregiver-based resources, including those that provided “better information/resources available to help caregivers better understand the impact of allergies” and support to help the caregiver “understand the context/drivers of the [child’s] behaviours”.

Specific allergy management for children, including access to oral food challenges, specialist staff and specific allergy diagnosis were reported as a strength by some participants (*n* = 11), with one participant reporting they were able to support the “family with generalised feeding education to build skills around increasing variety within safe foods [both as a sensory preference and a safe food from an allergy perspective]”. Other participants reported they needed “less wait time for immunology/allergy review and food challenges”. Some participants (*n* = 3) reported wanting better access to allergy testing with one reporting the “wait time is VERY long”. Another participant reported that they would like to have the “ability to test for IgE-mediated food allergies with an aim to ‘de-label’ patients who do not actually have an allergy [anymore]”.

### 3.7. Resources

Limited access to different staff disciplines, appropriate levels of staffing, and multidisciplinary clinics were reported as a human resource weakness by some participants (*n* = 11). It was noted that within the hospital system, more effective access to specialist medical staff and multidisciplinary teams was described. One participant reported that they had “dedicated medical teams to assess and diagnose food allergies”, and another had “paediatricians specifically for feeding issues [who] work very closely with a team of dietitians”.

The availability of psychology services for these families was also raised as a concern across both hospital (*n* = 34) and non-hospital (*n* = 43) settings, with one participant stating, “I feel all parents/carers of children with feeding difficulties would benefit from psychological support”. Many participants mentioned that in the perfect-world scenario, psychology (*n* = 31) and dietetic (*n* = 32) services would specifically be on their “wish list” to support this population.

With regard to clinical resources, the use of good parent-reported outcome measures (PROMs) and access to instrumental assessments were reported by four participants as being strengths of their services, with one participant reporting their service was “PROM focused and driven”. Others, however, reported that they felt they required “up-to-date strategies to support feeding behaviours” and “more specific guidelines for treating these children”. Participants suggested that “resources with food ideas that are appropriate target foods/play foods [that are allergy-safe]” and “better access to variety of foods in clinic” would be valuable.

Staff training in both food allergy management and paediatric feeding disorder was highlighted as an area of need. Overall, 13 participants, most of whom were from non-hospital settings (*n* = 10), highlighted the lack of knowledge as an issue requiring “more knowledge/education around allergies/intolerances”, and “more training in food allergies and how to manage feeding difficulties in this context”. Having experienced staff, however, was reported as a strength by 12 participants, who described that “…we have a number of experienced team members who manage the most challenging cases”, with others reporting staff with “extensive knowledge and experience”.

## 4. Discussion

This survey investigated service availability and perceptions of care by Australian health professionals managing children with food allergies and paediatric feeding disorder across a range of health settings. Speech pathologists were the largest professional group represented within the participants. The majority of participants worked in hospital-based services. Service delivery, intervention, and resources were the three categories coded from participant’s open-ended responses using content analysis. Concerns were raised by participants regarding timely access to specialist allergy and medical services as well as the availability of multidisciplinary feeding services outside of the hospital setting. The availability of psychology and dietetics services was identified as a gap for families, both within and outside of the hospital system. Clinical education for staff on both paediatric feeding disorder and food allergies, and the intersection between the two was also identified as an area of need.

Multidisciplinary care is considered best practice for children with allergies and also with for children with paediatric feeding disorder due to the multifaceted complexity of the two conditions [16,18,20,22]. Despite the recognition of the importance of coordinated multidisciplinary care, the current study found that many respondents reported a lack of coordinated multidisciplinary care for children presenting with both food allergies and paediatric feeding disorder. One of the main issues is the ability for families to access specialist care. It is well known that access to paediatric allergy care is an issue both in Australia [23] and internationally [16,20]. Limited access to these allergy services could result in unnecessary dietary restriction for children, affecting weight and growth, and limited wider exposure to foods that could support both the child’s feeding development and their overall nutritional profile [6]. This may then lead to ongoing and unnecessary stress and burden for caregivers [7]. To attempt to address this gap in care for children and families, the Australian Federal Government has funded the National Allergy Council and Australasian Society of Clinical Immunology and Allergy to improve access to quality care for food allergies and to scope and develop allergy-related education initiatives for health professionals, including those in primary care and allied health [36]. As for allergies, gaining access to specialist services for paediatric feeding disorder in Australia is complicated [28], and services can be fragmented and confusing, particularly for families with low health literacy [27]. Services are affected by long waiting times [27,28] and children with more mild feeding concerns or those who are not medically complex often fall outside the public service eligibility criteria [27]. More research into appropriate multidisciplinary models of care is required to further reform allergy and feeding services.

Early intervention is valuable for both food allergies and paediatric feeding disorder. Timely access to allergy care is important to ensure appropriate dietary management and to avoid unnecessary elimination of different foods that can pose a nutritional risk to young children [16,37]. Also imperative is timely anaphylaxis management to reduce fear and anxiety for both caregivers and children and the chance of life-threatening events [16]. Paediatric feeding disorder is known to have wide-ranging impacts on both the child and their family [18,28], and timely access to early intervention can help reduce the associated conflict and stress [37,38]. Having positive early feeding experiences can help support the bond between caregivers and children [39]. However, many children are diagnosed with food allergies during infancy [20], which can result in early adverse reactions to food and limited exposure to a variety of foods [6]. This can then lead to disruptions in positive mealtime experiences between caregiver and child [6,20,37]. It is anticipated that more timely feeding intervention for children with food allergies could minimise the risk of paediatric feeding disorder [37]. This study, however, has identified that early intervention for both allergy and feeding services for Australian families is often not timely, particularly when external to hospital settings.

Despite the identified concerns regarding caregiver mental health for families of children with food allergies [7,17,40,41,42,43] and paediatric feeding disorder [44,45,46], most of the health professionals surveyed in the current study reported limited access to any mental health or caregiver supports for this group. This finding is consistent with mental health access in allergy services in the UK [47]. Caring for a child with both food allergies and paediatric feeding disorder presents a significant and ongoing burden for caregivers, with potentially heightened emotional, physical, and cognitive demands. Caregivers must navigate the daily fear of allergen exposure and its potentially unpredictable and life-threatening consequences [8,48], while also managing concerns related to their child’s feeding skill development, weight gain, growth, and challenging mealtime behaviours [8,20,37]. Given the significant mental health implications, considering caregiver needs in designing future services is critical.

Health professionals’ knowledge of food allergies and/or paediatric feeding disorder was also identified as lacking for many participants. Food allergy has previously been identified as a training need for allied health staff in the UK and Europe, and in Australia [22]. Increased training needs for health professionals in paediatric feeding disorder have also been identified [29,30], particularly for the management of more complex cases [27]. The combination of food allergies and paediatric feeding disorder in the one child potentially presents an even more complex clinical picture for clinicians providing their care. Improved health professionals’ knowledge using evidence-based training allows for more consistent clinical practice and less confusion for caregivers navigating these services. This in turn supports health professionals to empower families in their own self-management and coping abilities, which are known to have an impact on quality of life [16,22]. To ensure the outcomes for the child’s feeding are optimised, further research into the specific training needs of health professionals supporting families of children with food allergies and paediatric feeding disorder would be of benefit.

## 5. Limitations

It is acknowledged that this was a small survey that mostly included speech pathologists, and participants from New South Wales, Australia, which may have impacted the generalisability of results. Additionally, many public health care services in Australia are provided free of charge, particularly for children [49] and some services may be funded via the National Disability Insurance Scheme [50] for eligible families. Differences in health systems in other countries should be considered in applying these results to local practice. It is also acknowledged that this survey only captured the opinions of health professionals, and caregivers were not surveyed directly. Further research is required to document the impact on caregivers and explore their perceptions on care and services. Despite these limitations, this is the first known survey to capture health professionals’ perspectives regarding service options for children with food allergies and paediatric feeding disorder.

## 6. Conclusions

Australian health professionals were surveyed to gain insights into the clinical services available for children with food allergies and paediatric feeding disorder, and their families. Participants reported concerns regarding the lack of timely access for families to a multidisciplinary team, particularly outside of hospital settings. This included reports of limited access to mental health professionals for children and families. A need for further training in this area for clinicians was described. Further research into co-designed, multidisciplinary early intervention and clinician training that supports improvements in care for these children and provides insight into the perspectives of caregivers is recommended.

## Figures and Tables

**Table 1:** Participant demographics (*n* = 98).

Characteristic	*n* (%)
*Discipline*	
Speech Pathologist	39 (40)
Paediatrician	6 (6)
Dietitian	22 (22)
Occupational Therapist	3 (3)
Nurse/CNC/CNS/Nurse Practitioner	21 (21)
GP	3 (3)
Allergist/Clinical Immunologist	2 (2)
Other (AHA, PT)	2 (2)
*Work Location*	
New South Wales	52 (52)
Queensland	24 (24)
Tasmania	2 (2)
Victoria	8 (8)
Western Australia	6 (6)
Northern Territory	1 (1)
South Australia	8 (8)
Australian Capital Territory	1 (1)
Telehealth	1 (1)
Unknown	1 (1)
*Work Setting*	
Tertiary Hospital	36 (37)
Non-Tertiary Hospital	15 (15)
Hospital Inpatient/Outpatient	55 (56)
Community Health	25 (25)
Specialist Allergy Practice	3 (3)
MDT Feeding Clinic	10 (10)
Early Childhood Nurse/Primary Health Care	3 (3)
GP Practice	2 (2)
Private Practice	22 (22)
*Time Working with Paediatric Caseload*	
<1 yr	1 (1)
1–5 yrs	10 (10)
6–10 yrs	25 (26)
11–19 yrs	34 (35)
20+ yrs	27 (28)

CNC = clinical nurse consultant; CNS = clinical nurse specialist; GP = General Practitioner; AHA = allied health Assistant; PT = physiotherapist; MDT = Multidisciplinary Team.

**Table 2:** Participant caseload details (*n* = 98).

	*n* (%)
*Age of caseloads*	
Infant 0–12 mths	73 (74)
Toddler 1–3 yrs	82 (84)
Preschool 3–5 yrs	73 (74)
Primary School 5–12 yrs	40 (41)
High School 12–18 yrs	18 (18)
*Percentage of caseload presenting with feeding concerns*	
<10%	30 (31)
11–50%	41 (42)
>50%	27 (27)
*Percentage of caseload with feeding concerns and food allergies*	
<10%	34 (35)
11–50%	50 (51)
>50%	14 (14)
*Most common comorbidities reported in clients with feeding concerns*	
Autism spectrum disorder	85 (87)
Food allergies	82 (84)
Global developmental delay	72 (73)
History of prematurity	68 (70)
Sensory processing concerns	66 (67)
Anxiety disorders	65 (66)
Eosinophilic oesophagitis	57 (58)
Complex medical needs	55 (56)
Food protein-induced enterocolitis	53 (54)
Tube dependency	53 (54)
Genetic disorders	50 (51)
*Most common feeding concerns/difficulties reported by participants*	
Won’t try new foods	76 (77)
Child has self-restricted diet	68 (70)
Caregiver reported stress	65 (67)
Child has poor weight/growth	56 (59)
Child refuses to eat	44 (45)
Gagging/vomiting	41 (42)
Child showing signs of anxiety at meals	39 (40)
Caregiver is making multiple options for each meal	34 (35)
Child is crying/having tantrums during the meal	26 (26)

**Table 3:** Categories and subcategories from content analysis with example quotes.

Category	Subcategory	Example Quotes
Service delivery	Models of service provision	“Regular reviews.” “Flexible appointments.” “…[we provide] intensive support.” “No feeding therapy blocks provided.” “Only able to provide an assessment and limited short-term intervention…with limited ability to provide ongoing or long-term support.”
	Comprehensive care	“Multidisciplinary feeding clinic with speech pathologist, dietitian, medical and psychological input” “Good communication between…departments.” “…holistic MDT approach” “Home visits.” “Improved access to specialised feeding assessment clinics…” “Face-to-face contact would likely enhance services.”
	Referrals	“Established links with medical, nursing and allied health that can facilitate timely intervention.” “Able to access specialist input fairly easily (e.g., immunologist, gastroenterologist).” “Easier access to specialists.” “Less wait times for immunology/allergy review and food challenges.” “…local community health follow up.” “….and minimal services in the community to link into.”
Intervention	Feeding intervention	“…therapy with child and family—hands-on and practical” “…range of intervention models.” “The ability to offer more intensive service for management and therapy.”
	Intervention directed at caregivers	“…initial education around the relationship between food allergy and feeding difficulties/behaviours” “Listen to the parent, educate, empower them and refer as needed.” “…including parenting support as well as psychological support (assist with anxiety).” “Parent support groups and regular parent education sessions.” “Better information/resources available to help parents understand [the] impact of allergies.”
	Allergy intervention	“…allergy specific diagnosis, oral food challenges/plans” “…clarifying what foods definitely should not be eaten (due to allergy) therefore (hopefully) broadening options for other foods to be tried.” “Ability to test for IgE-mediated food allergies in order to de-label patients who do not actually have an allergy.” “Would love to expand oral food challenges to de-label allergies where appropriate.”
Resources	Human resources	“Access to feeding specialists for additional support or guidance.” “Able to provide MD feeding [multidisciplinary feeding] team support for children.” “Access to a range of health professionals due to tertiary setting.” “Solid understanding/knowledge across department.” “It’s impossible to provide a comprehensive service with the resources we have.” “…more staff to manage the load.” “More funding for more service.” “Myself and the dietician are the only people supporting feeding for an area larger than Victoria.” “There are not enough services to meet demand resulting in clinicians not making referrals when a family really does need support.”
	Clinical resources	“…availability to instrumental investigations as needed (e.g., VFSS/FEES).” “…and ongoing protocols.”
	Access to training	“We have excellent peer supervision to manage community clients.” “More knowledge/education around allergies, tolerances etc.” “….courses can be very expensive for staff to fund themselves.”

## Data Availability

The data presented in this study are available on request from the corresponding author to maintain the confidentiality of the participants and health services involved.

## Supplementary Materials

The following supporting information can be downloaded at https://www.mdpi.com/article/10.3390/children12070905/s1.
