# Supplementary material for: Current Practices and Recommendations for Children with Food Allergies and Feeding Behaviours: Insights from a Survey Among Australian Health Professionals
Source: Children (Basel). 2025 Jul 9;12(7):905. doi: 10.3390/children12070905 (PMC12293343; doi:10.3390/children12070905)
Supplement: Supplementary file 1 [file children-12-00905-s001.zip › children-3708519-supplementary.pdf]

THE UNIVERSITY OF QUEENSLAND
AUSTRALIA
CREATE CHANGE

Feeding Behaviours and characteristics in Young Children with Food Allergies

Survey Questions for Health Professionals:

DEFINITIONS: Please use the following definitions as you complete the survey.

• **Food Allergy:** any IgE-mediated (e.g. anaphylaxis, general food allergy) or non-IgE-mediated food allergy (e.g. eosinophilic oesophagitis – EOE, Food Protein Induced Enterocolitis Syndrome – FPIES).

• **Feeding difficulties:** any medical, anatomical, or behavioural difficulty relating to mealtimes and/or the physical act of eating and drinking.

• **Challenging feeding behaviours**: any behaviour that disrupts the feeding context or affects the caregiver-child relationship associated with feeding.

• **Paediatric Feeding Disorder:** "impaired oral intake that is not age appropriate, and is associated with medical, nutritional, feeding skill and/or psychosocial dysfunction" (Goday et al., 2019, p. 125)

DEMOGRAPHICS:

1. **What is your profession?**

Speech Pathologist | Paediatrician/ Specialist | Dietitian | Occupational Therapist | Nurse/CNC/CNS/ Nurse Practitioner | Psychologist | GP | Allergist immunologist | Gastroenterologist | Other

2. **What is your main state of work?**

Western Australia | South Australia | Victoria | New South Wales

Queensland | Northern Territory | Tasmania | ACT

3. **What is your clinical workplace context?** (Please tick all relevant contexts)

Tertiary Hospital | Non-tertiary Hospital | Hospital Inpatient | Hospital Outpatient | Community Health | Specialist Allergy Practice | Multidisciplinary Feeding Clinic

Early Childhood/ Primary Healthcare Clinic | GP Practice | Private Practice | Non Government Organisation | Disability Sector | Sole Clinician | Other – please specify

4. **How long have you been working in your profession?**

< 1 year | 1-5 years | 6-10 years | 11-19 years | 20+ years

5. **How long have you been working in paediatrics?**

< 1 year	1-5 years	6-10 years	11-19 years	20+ years

6. **Which age group makes up the <u>majority</u> of the children you see?**

Infant/Baby 0-12months	Toddler 1-3 yrs	Preschool 3-5yrs	Primary School Aged 5-12ys	High School Aged 12-18yrs

7. What are some of the reasons children are referred into your service? (Open response).

<u>CLINICAL CASELOAD – FEEDING DIFFICULTIES:</u>

1. **How long have you been working with children who present with feeding difficulties?**

< 1 year	1-5 years	6-10 years	11-19 years	20+ years

2. **How confident do you feel in your clinical skills in managing a child with feeding difficulties?**

Advanced (I'm pretty confident and can teach others)	Intermediate (I have some experience but still seek supervisory support for difficult cases)	Foundation (I have done some observations and I know a little bit)	Novice (I know next to nothing in this area)

3. **Skip logic only – if Advanced chosen How long had you considered yourself to be a paediatric feeding specialist**

< 1 year	1-5 years	6-10 years	11-19 years	20+ years

4. **Which age group makes up the majority of the children you see with feeding difficulties?** (Select all that apply)

Infant/Baby 0-12months	Toddler 1-3 yrs	Preschool 3-5yrs	Primary School Aged 5-12ys	High School Aged 12-18yrs

5. **What other comorbidities do you see in your feeding caseload?**

Autism Spectrum Disorder	Global Developmental Delay	Genetic syndromes	Eosinophilic Oesophagitis	Food Protein Induced Enterocolitis Syndrome	Complex medical needs
Tube dependency	Food Allergies	History of Prematurity	Other – please specify		

6. **Approximately what percentage of children on your caseload present with feeding difficulties?**

0-10%	11-30%	31-50%	51-75%	76-100%

<u>*DEFINITION REMINDER:</u> Please continue to use the following definitions as you complete the survey.**

• **Food Allergy: any IgE-mediated** (e.g. anaphylaxis, general food allergy) or non-IgE-mediated food allergy (e.g. eosinophilic oesophagitis – EOE, Food Protein Induced Enterocolitis Syndrome – FPIES).

• **Feeding difficulties:** any medical, anatomical, or behavioural difficulty relating to mealtimes and/or the physical act of eating and drinking.

• **Challenging feeding behaviours:** any behaviour that disrupts the feeding context or affects the caregiver-child relationship associated with feeding.

• **Paediatric Feeding Disorder:** "impaired oral intake that is not age appropriate, and is associated with medical, nutritional, feeding skill and/or psychosocial dysfunction" (Goday et al., 2019, p. 125)

ASCIA Website for further information: https://www.allergy.org.au/hp/papers

7. **Approximately what percentage of children on your caseload with feeding difficulties ALSO present with reported challenging feeding behaviours or behaviours around mealtimes and food?**

   0-10%   11-30%   31-50%   51-75%   76-100%

8. **Approximately what percentage of children on your caseload with feeding difficulties ALSO present with food allergies or a history of food allergies?**

   0-10%   11-30%   31-50%   51-75%   76-100%

9. **From the list below, pick the top 5 challenges reported by parents of children with feeding difficulties AND food allergies on your caseload?**

Child won't try new foods	Parental reported stress
Child refuses to eat	Generalised behaviour issues reported for the child
Child is refusing to come to the mealtime at all	School refusal
Child is leaving the table/getting up and down from table during the meal	Child reports difficulties swallowing food/drink
Child is crying/having tantrums at mealtime	Gastrointestinal issues
Child is gagging/vomiting	Abdominal pain
Child self-restricting diet	Child had poor weight gain/growth
Child shows signs of anxiety	Issues with accessing services
Parent is making multiple options for each meal	Constipation/Diarrhoea

10. **Are there any other concerns (not listed above) that parents report regarding challenging behaviours at mealtimes?** (Free text)

HEALTH SERVICE CONTEXT:

1. **What services do you have access to WITHIN your clinical context to refer children with feeding behaviours AND a food allergy? (select all that apply)**

Speech Pathologist	Paediatrician/ Specialist	Dietitian	Occupational Therapist	Nursing support	Psychologist	GP
Gastroenter ologist	Allergist/ Immunologist	Unsure	Other			

2. **What services do you have access to OUTSIDE of your clinical context to refer children with feeding behaviours AND a food allergy? (select all that apply)**

Speech Pathologist	Paediatrician/ Specialist	Dietitian	Occupational Therapist	Nursing support	Psychologist	GP
Gastroenter ologist	Allergist/ Immunologist	Unsure	Other			

3. **What does your service do well in providing support for children who present with feeding behaviours AND food allergies?** (Free text).

4. **What needs to be improved in your service to providing support for children who present with feeding behaviours AND food allergies?** (Free text).

5. **In an ideal world, what would you include in a service for children who present with feeding behaviours AND food allergies?** (Free text).
